# Multiple geochemical factors may cause iodine and selenium deficiency in Gilgit-Baltistan, Pakistan

**DOI:** 10.1007/s10653-021-00936-9

**Published:** 2021-04-24

**Authors:** Saeed Ahmad, Elizabeth H. Bailey, Muhammad Arshad, Sher Ahmed, Michael J. Watts, Scott D. Young

**Affiliations:** 1 Division of Agricultural and Environmental Sciences, School of Biosciences, University of Nottingham, Sutton Bonington Campus, Loughborough, LE12 5RD Leicestershire UK; 2 Mountain Agriculture Research Centre Gilgit, Pakistan Agricultural Research Council), Gilgit-Baltistan, Pakistan; 3 Centre for Environmental Geochemistry, Inorganic Geochemistry, British Geological Survey, Nottingham, NG12 5GG UK

**Keywords:** Iodine, Selenium, Soil, Water, ICP-MS

## Abstract

Deficiencies of the micronutrients iodine and selenium are particularly prevalent where populations consume local agricultural produce grown on soils with low iodine and selenium availability. This study focussed on such an area, Gilgit-Baltistan in Pakistan, through a geochemical survey of iodine and selenium fractionation and speciation in irrigation water and arable soil. Iodine and selenium concentrations in water ranged from 0.01–1.79 µg L^−1^ to 0.016–2.09 µg L^−1^, respectively, which are smaller than levels reported in similar mountainous areas in other parts of the world. Iodate and selenate were the dominant *inorganic* species in all water samples. Average concentrations of iodine and selenium in soil were 685 µg kg^−1^ and 209 µg kg^−1^, respectively, much lower than global averages of 2600 and 400 µg kg^−1^, respectively. The ‘reactive’ fractions (‘soluble’ and ‘adsorbed’) of iodine and selenium accounted for < 7% and < 5% of their total concentrations in soil. More than 90% of reactive iodine was organic; iodide was the main inorganic species. By contrast, 66.9 and 39.7% of ‘soluble’ and ‘adsorbed’ selenium, respectively, were present as organic species; inorganic selenium was mainly selenite. Very low distribution coefficients (kd = adsorbed/soluble; L kg^−1^) for iodine (1.07) and selenium (1.27) suggested minimal buffering of available iodine and selenium against leaching losses and plant uptake. These geochemical characteristics suggest low availability of iodine and selenium in Gilgit-Baltistan, which may be reflected in locally grown crops. However, further investigation is required to ascertain the status of iodine and selenium in the Gilgit-Baltistan food supply and population.

**Supplementary Information:**

The online version contains supplementary material available at 10.1007/s10653-021-00936-9.

## Background

Iodine (I) concentration in the environment is highly variable (Wu et al., 2013). Unlike most other elements, weathering of rocks and sediments is not a major source of I for the soil–plant system (Johnson, 2003). Only a small proportion of the soil I available to plants is derived directly from rock weathering (Fuge & Johnson, 2015; Jensen et al., 2019). In contrast, oceans are major reservoirs of I (Fuge & Johnson, 2015; Manousou et al., 2019; Medrano-Macías et al., 2016) and volatilisation from ocean water and movement through the atmosphere plays an essential role in I cycling through the environment and the biosphere (Johnson, 2003; Medrano-Macías et al., 2016). Thus, I input from the atmosphere, as dry or wet precipitation, often contributes greatly to soil and plant I (Bowley et al., 2019; Fuge & Johnson, 1986; Jensen et al., 2019; Johnson, 2003). It is recognised that I concentrations are greater in coastal areas compared to inland and mountainous regions located away from coasts (Bowley et al., 2016; Fuge & Johnson, 2015; Humphrey et al., 2018). Apart from inputs, the concentration of I in soil is also affected by several factors affecting the retention capacity of the soil; these include climate, topography and soil characteristics such as organic matter concentration and pH (Bowley et al., 2019; Fuge & Johnson, 2015; Humphrey et al., 2018, 2020; Mohiuddin et al., 2019). Therefore, a soil with a large I concentration does not necessarily produce I-rich plants because of factors affecting the *availability* of soil I (Bowley et al., 2019; Fuge & Johnson, 1986; Mohiuddin et al., 2019). Fresh waters generally have low I concentrations (Fuge & Johnson, 2015; Johnson, 2003) unless rivers run through I-rich sedimentary rocks (Fuge, 1989; Moran et al., 2002), whereas groundwaters typically have higher I concentrations; values of up to 1890 µg L^−1^ have been reported in some areas (Li et al., 2013; Qian et al., 2017). Iodine is not considered essential for terrestrial plants; however, plants absorb I through their roots and leaves (Bowley et al., 2019; Medrano-Macías et al., 2016). The rate of I accumulation differs between plants (Hong et al., 2007; Whitehead, 1984). For example, Hong et al., (2007) in their study on I accumulation in various vegetables reported that I accumulation rate varied in vegetables in the order: pakchoi > celery > radish > capsicum.

Selenium (Se) is among the most widely distributed elements in the Earth’s crust and is mainly associated with sulphide minerals (Dhillon et al., 2019; Johnson et al., 2010; Wang et al., 2016). Weathering of rocks is one of the primary sources of Se in soil (Fordyce, 2013; Saha et al., 2017; Shamberger, 1981). The majority of rocks contain low concentrations of Se; therefore, Se-deficient soils are more common than ‘seleniferous’ soils (Fordyce, 2013). Selenium concentration in most soils ranges from 0.01 to 2.0 mg kg^−1^ although some seleniferous soils have Se concentration up to 1250 mg kg^−1^ (Dhillon et al., 2019; Fordyce, 2013; Fordyce et al., 2010; Winkel et al., 2012). Most surface waters have small concentrations of Se (0.06–0.12 µg L^−1^), whereas groundwater Se can reach up to 6000 µg L^−1^ in some areas (Alexander, 2015; Fordyce, 2013), presumably because of Se-rich parent material. Selenium is not considered essential for higher plants, but it is taken up by plants via sulphate (selenate) and phosphate (selenite) transporters (Alexander, 2015; Gupta & Gupta, 2017; Malagoli et al., 2015; Riaz et al., 2018; White, 2016). Plants differ in their ability to accumulate Se (Alexander, 2015; Broadley et al., 2006; Barillas et al., 2011; Ebrahimi et al. 2019; Woch & Hawrylak-Nowak, 2019; Yang et al. 2017). For example, Brassica species (rapeseed, broccoli, cabbage), allium spices (onion and garlic) and Brazil nuts accumulate higher concentration of Se compared to grasses and grains (wheat, oats, rye and barley) (Alexander, 2015; Yang et al., 2017).

Both I and Se are important micronutrients for human and animal health, and their deficiency or toxicity can result in serious health complications. Deficiencies can be resolved by supplying sufficient I and Se in the diets of affected populations via food fortification with I and Se or use of iodised salt (Hetzel, 1983; Lyons, 2018; Malagoli et al., 2015; Rayman, 2000; Sun et al., 2017;). Iodine deficiency is generally widespread in remote mountainous areas (Faridullah, 2017; Kelly & Snedden, 1958). Thus, Gilgit-Baltistan, located in the Himalayan region, has a history of I deficiency disorders (IDD) (Stewart, 1990; Shah et al., 2014; Faridullah et al., 2017; Khattak et al., 2017). Furthermore, co-existing deficiency of I and Se can result in some extreme forms of IDD (e.g. cretinism) (Eastman & Zimmermann, 2018; Lyons, 2018; Vanderpas et al., 1990); incidence of cretinism has been historically reported in Gilgit-Baltistan (Stewart, 1990). The population of Gilgit-Baltistan largely consume locally grown agricultural produce (Mountain Agriculture Research Centre, personal communication, December 2019), and there is limited data available on the status of environmental I and Se in Gilgit-Baltistan.

This study aimed to assess the status of I and Se in Gilgit-Baltistan, focussing on factors controlling I and Se concentration and speciation in soils and water and their potential availability to plants.

## Methods

### Study sites

**Fig. 1:**
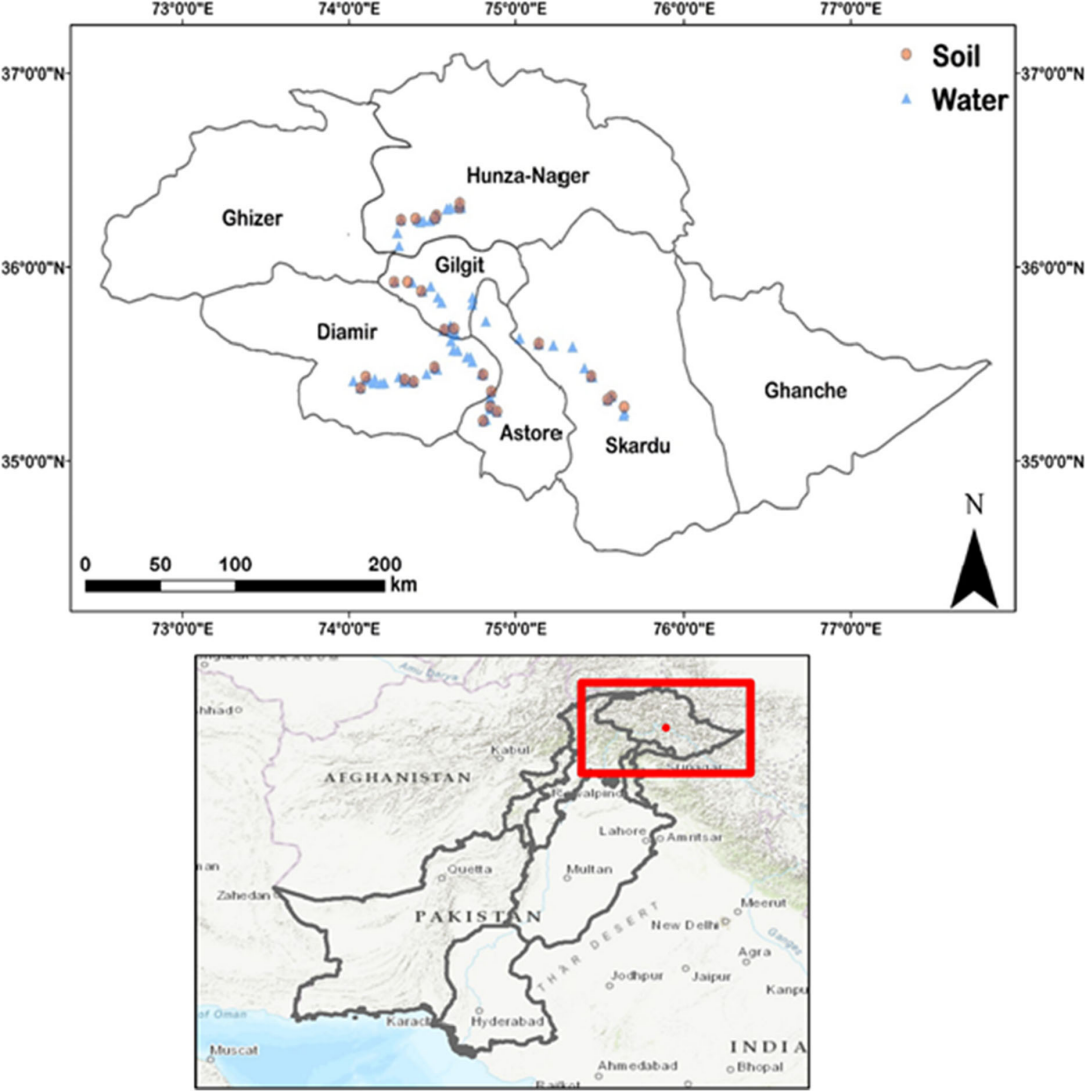
Sampling locations in Gilgit-Baltistan

Study sites were selected in five districts of Gilgit-Baltistan, which were chosen based on their accessibility and the availability of fertile agriculture land; the sites were identified with the help of colleagues from the Mountain Agriculture Research Centre (MARC). The sampling districts included Gilgit, Diamer, Hunza-Nagar (Hunza-N), Astor and Skardu (Fig. 1). Overall, twenty-six villages were surveyed with five villages from each district apart from Hunza-N where six villages were sampled. At the time of planning this project, Hunza-N was one district, but it was later divided into district Hunza and district Nagar; in this study, we have referred to Hunza-N as one district. All the sampling sites were located within the altitude range 1000 to 2700 m above sea level and approximately 1400 km from the nearest coast.

### Sample collection and processing

Samples were collected in July and August of 2016. Water was sampled in the villages identified for soil sampling and also between sample villages. A total of 66 irrigation water samples were collected from surface water sources including rivers, streams and lakes. Water samples were filtered at the point of sampling, using syringe filters (< 0.22 µm) and kept in the dark during transportation to the MARC laboratory in Gilgit where they were stored at 4ºC pending shipping to the University of Nottingham for elemental analysis by ICP-MS.

A composite topsoil (0–20 cm) sample was collected with a stainless-steel auger from arable land in each village. The soil was air-dried and sieved (< 2 mm) at the MARC laboratory and then shipped to the University of Nottingham where a sub-sample of 10 g was finely ground in an agate ball mill (Retsch PM400, Haan, Germany) for elemental analysis.

### Sample characterisation and chemical analysis

#### Water

Water pH and electric conductivity (EC) were measured with a portable pH and EC meter (HANNA HI-98129) at the source. Dissolved inorganic carbon (DIC) and total carbon (TC) in water samples were measured on a Shimadzu TOC-VCPH coupled with an ASI-V unit (Shimadzu UK Ltd) after diluting the water sample (10 mL sample + 10 mL Milli-Q water (18.2 MΩ cm)) following Karim (2018). A range of concentrations (10 to 50 mg L^−1^) of potassium hydrogen phthalate (C_8_H_5_KO_4_) were used to calibrate the instrument for TC analysis, while Na_2_CO_3_ (10–50 mg L^−1^) was used for DIC calibration. Dissolved organic carbon (DOC) was determined by difference (TC – DIC). Selenium and I analysis were undertaken using a single quadrupole ICP-MS (model iCAP-Q, Thermo Scientific, Bremen, Germany) on samples preserved in 2% HNO_3_ and 1% tetra methyl ammonium hydroxide (TMAH), respectively.

#### Soil

Soil pH was measured using a pH meter (HANNA, model pH 209) with combined glass electrode on a soil–water suspension of 5 g soil (< 2 mm sieved) and 12.5 mL Milli-Q water after shaking on an end-over-end shaker for 30 min (Rowell, 1994). Mechanical analysis (soil texture) was undertaken by laser granulometry to determine clay (< 4 µm), silt (≥ 4 µm and ≤ 63 µm) and sand (> 63 µm) particles; the grain size < 4 µm was used to define clay particles following Kerry et al., (2009). The finely ground soil was used for measuring concentrations of total carbon and inorganic carbon in an Elemental Analyser (Model Flash EA1112, CE Instruments) and Shimadzu TOC-VCPH coupled with an SSM-5000A solids module (Shimadzu UK Ltd), respectively, following Mathers (2015) and Ligowe et al., (2019).

Oxides of Fe, Mn and Al in soil samples were determined in citrate-bicarbonate-dithionate (CBD) extracts of finely ground soil by ICP-MS following Mathers (2015) and Ligowe et al., (2019). Soil total I (I_T_) was extracted with 10% TMAH which was diluted to 1%, after centrifugation (2500 g), for analysis by ICP-MS as described in Watts and Mitchell (2009). Acid digestion (HNO_3_-HClO_4_-HF) of finely ground soil was undertaken in PFA vessels using a teflon-coated graphite block digester (Model A3, Analysco Ltd) controlled by a Eurotherm temperature control unit following Mathers (2015), Karim (2018) and Sanders (2018). Soil digests were diluted in Milli-Q water prior to analysis of total selenium (Se_T_) concentration by ICP-MS.

A three-stage sequential extraction of < 2 mm sieved soil was undertaken with potassium nitrate (0.01 M KNO_3_) followed by potassium dihydrogen phosphate (0.016 M KH_2_PO_4_) to determine ‘soluble’ and ‘adsorbed’ fractions of I and Se following Karim (2018) and Ligowe et al., (2020). This was followed by extraction with 10% TMAH to determine ‘Organic’ fractions of Se and I. Speciation analysis of I and Se were undertaken on the KNO_3_ and KH_2_PO_4_ extracts of soil samples using an HPLC unit (Dionex ISC-5000) coupled to the ICP-MS as mentioned in Bowley et al., (2016), Karim (2018) and Sanders (2018). The chromatography eluent consisted of 4.00 g L^−1^ NH_4_NO_3_, 20 mLl L^−1^ methanol, 0.00325 g L^−1^ NH_4_-EDTA and 12.1 g L^−1^ Tris buffer. The stationary phase used was a Hamilton PRP X-100 anion exchange column (100 × 4.1 mm; 5 µm particle size); the eluent flow rate was 1.4 mL min^−1^. Working standards of 5 µg L^−1^ I and Se reduced species (I^−^ and Se^IV^) and oxidised species (IO_3_^−^ and Se^VI^) were run between the samples at regular intervals to enable correction for instrumental drift. The peaks obtained for each sample were manually integrated using Chromeleon^TM^ software, and then, peak areas were converted to concentration in Microsoft Excel 2016 by considering the peak area of standards (supplementary information Figs A1 and A2) as reference. Species determined included iodide, iodate, selenite and selenate; organic I and Se species were calculated by difference from the total I and Se concentrations in the ‘soluble’ and ‘adsorbed’ fractions.

**Fig. 2:**
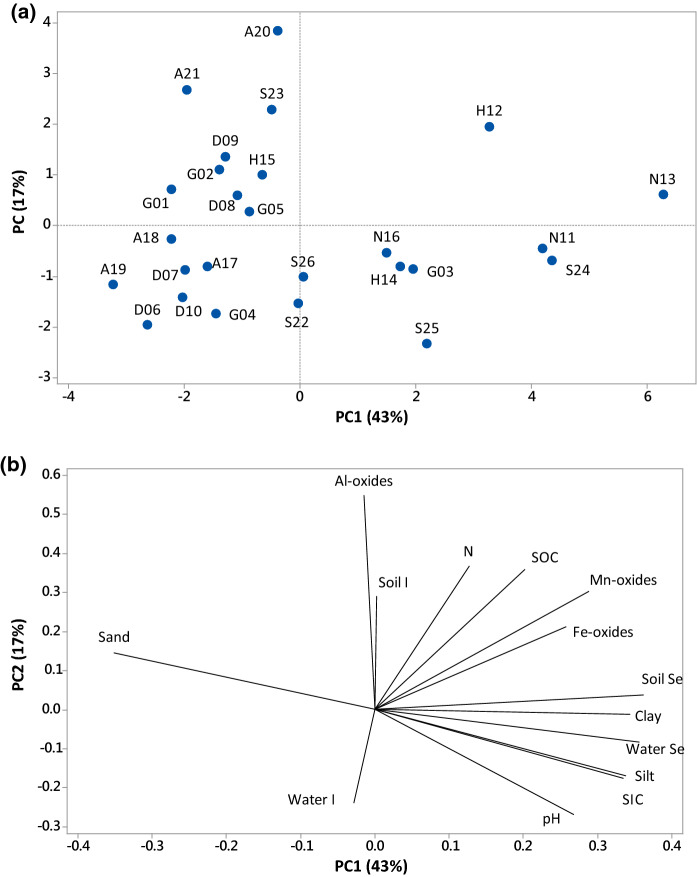
Principal component analysis results of iodine and selenium in soil **a** location of soil samples, **b** parameters affecting iodine and selenium concentration in soil

### Distribution coefficient of iodine and selenium in soil

The distribution coefficient (kd) is the ratio of adsorbed fraction to soluble fraction and was calculated both for I and Se, respectively, from Eq. 1.

1 $${\text{kd}} = \frac{{C_{{{\text{ads}}}} }}{{C_{{{\text{sol}}}} }}$$

where C_ads_ and C_sol_ are the concentrations of soil I or Se (µg kg^−1^) in the adsorbed and soluble fractions.

### Quality control and quality assurance

All sample preparation and analysis procedures were undertaken with replication; generally, replicates were within ± 1% for individual samples. Operational blanks (OBs) were run within each batch of analysis to correct for contamination associated with the sample preparation and analytical procedures. The OBs were also used to estimate limits of detection (3 × standard deviation of 10 × OBs). A soil certified reference material (CRM) (Montana soil – NIST 2711a) was used for quality assurance of the elemental analysis. The average recovery of Se in the CRM was within ± 10% of the reported values. Calibration solutions of I and Se were always run prior to and during sample analysis by ICP-MS; internal standards were used to correct for drift (Rh in acidic matrices, Re in TMAH).

### Statistical analysis

Basic statistical calculations including mean, median, standard error and standard deviation were performed in Microsoft Excel 2016. Pearson’s correlation analysis, analysis of variance (ANOVA) and principal component analysis (PCA) were performed in Minitab (version 18.1). Pearson’s correlation was used to describe association between analytes and various characteristics of water and soil samples, while ANOVA was applied to determine whether there was any significant difference between data from different locations. A* p* value of < 0.05 was considered significant.

## Results and discussion

### Water characteristics

The basic characteristics of all water samples are provided in supplementary information (Table B1). The majority of water characteristics, with the exception of pH, did not show significant variation among districts (*p* > 0.05). The median pH of samples was 7.9 and ranged from 7.0 to 9.2. Water EC was less than 1.0 dS m^−1^ in all samples with mean and median values of 0.210 and 0.173 dS m^−1^, respectively. Water with EC < 0.75 dS m^−1^ is suitable for irrigation and does not have any detrimental effect on plant growth (Bortolini et al., 2018; Zaman et al., 2018). Dissolved inorganic carbon (DIC) accounted for a large proportion (> 85%) of total carbon compared to dissolved organic carbon (DOC). Median DIC and DOC concentrations were 10.5 and 1.38 mg L^−1^, respectively, in all samples. The hardness of water, calculated as the apparent concentration of CaCO_3_, ranged from 21.1 mg L^−1^ to 328 mg L^−1^ in all water samples; concentrations of CaCO_3_ ≤ 60 mg L^−1^, 61–120 mg L^−1^, 121–180 mg L^−1^ and more than 180 mg L^−1^ are categorised as soft, moderately hard, hard and very hard, respectively (McGowan, 2000; USGS 2021). The sodium adsorption ratio (SAR) in all water samples was < 1 which means there is unlikely to be a problem with soil sodicity; values of SAR < 3 are suitable for a wide range of crops and unlikely to cause any soil health problems (Bortolini et al., 2018).

**Table 1:** Basic characteristics of soil samples from all sampling districts (BDL: below detection limit)

District	Sample code	pH	SIC	SOC	Sand	Silt	Clay	^1^Fe-ox	^2^Mn-ox	^3^Al-ox	^4^Comb-ox
			%					(g kg^−1^)			
Gilgit	G01	7.19	BDL	0.983	79.1	18.7	2.25	3.33	0.119	0.322	3.77
	G02	7.77	0.008	1.52	74.2	22.7	3.12	3.29	0.097	0.349	3.73
	G03	8.06	0.525	1.48	55.4	39.8	4.87	2.94	0.162	0.250	3.35
	G04	7.22	0.006	0.661	54.3	42.8	2.89	1.92	0.055	0.250	2.23
	G05	7.43	0.001	1.37	67.5	29.8	2.68	4.08	0.078	0.268	4.42
Diamer	D06	7.90	0.040	0.976	81.0	16.4	2.62	2.04	0.073	0.197	2.31
	D07	7.70	BDL	0.841	74.4	23.3	2.31	2.38	0.055	0.247	2.68
	D08	7.93	0.024	0.989	72.5	23.8	3.71	3.91	0.137	0.302	4.35
	D09	7.21	0.007	1.90	74.4	21.3	4.26	3.59	0.130	0.266	3.99
	D10	7.89	0.002	1.04	70.9	25.6	3.55	2.38	0.071	0.223	2.67
Hunza-N	N11	8.26	1.41	1.45	59.4	34.9	5.68	6.68	0.160	0.276	7.11
	H12	7.87	0.686	2.75	61.7	33.8	4.54	7.27	0.184	0.294	7.75
	N13	8.19	0.691	2.65	36.7	51.3	11.9	3.89	0.192	0.261	4.35
	H14	8.28	0.469	1.94	63.9	33.0	3.06	4.82	0.084	0.232	5.14
	H15	7.87	0.137	2.46	76.6	21.8	1.59	4.20	0.077	0.257	4.54
	N16	7.92	0.963	2.77	60.5	35.9	3.51	3.24	0.090	0.186	3.52
Astor	A17	7.34	0.005	2.03	72.2	25.2	2.56	2.04	0.053	0.167	2.26
	A18	6.83	BDL	1.57	74.1	23.0	2.91	1.92	0.060	0.242	2.22
	A19	6.90	BDL	0.410	82.2	16.3	1.48	3.23	0.054	0.220	3.50
	A20	7.16	BDL	2.21	74.2	22.2	3.67	4.48	0.179	0.453	5.12
	A21	6.70	BDL	1.62	74.8	21.9	3.35	2.79	0.096	0.370	3.26
Skardu	S22	8.18	0.038	1.11	58.6	38.1	3.33	1.97	0.081	0.192	2.24
	S23	7.51	0.004	1.99	69.2	27.8	2.98	2.51	0.116	0.307	2.93
	S24	8.04	1.30	1.42	39.6	51.3	9.10	4.42	0.146	0.258	4.83
	S25	8.33	1.54	1.06	57.2	36.7	6.11	4.12	0.110	0.216	4.45
	S26	8.23	0.448	0.984	64.5	31.0	4.53	2.97	0.137	0.240	3.34

^1^Iron oxide, ^2^Manganese oxide, ^3^Aluminium oxide, ^4^Combined metal oxides of Fe, Mn and Al

### Iodine in water

#### Total iodine

Iodine concentration in all water samples (*n* = 66) ranged from 0.01 to 1.79 µg L^−1^ with a median of 0.20 µg L^−1^ (supplementary information Table B1). There was no significant difference in I concentration between districts (*p* > 0.05). The I concentrations observed in the current study are at the lower end of the global surface water concentration range (0.01–212 µg L^−1^) reported by Fuge and Johnson (2015). Values of I were also less than I concentration in water from other regions with similar mountainous topography, such as Kabul and Nangarhar Afghanistan with average values of 15.4 and 7.6 µg L^−1^, respectively (Watts & Mitchell, 2009); San Juan province in Argentina at an average 40.2 µg L^−1^ (Watts, 2010); Kilimajaro district in Tanzania with an average I concentration of 22.4 µg L^−1^ (Watts et al., 2019). However, the results for Gilgit-Baltistan are comparable with I concentrations (< 0.1 µg L^−1^) reported by Day and Powell-Jackson (1972) in the Himalayan region of Nepal. Multiple factors, such as distance from the sea, rainfall and underlying geology, affect I concentration in fresh water (Fuge & Johnson, 2015). The nearest coast to the study area is at a distance of about 1400 km which reduces the chances of any marine influence in water I recharge. Furthermore, the area is located within a rain shadow and hence receives rainfall of only 254 mm per year. The geology is igneous or metamorphic in nature and highly variable (Malik & Azam, 2009). The low precipitation rates and the presence of igneous/metamorphic rocks are contributing factors to the low I concentrations in waters of the study area.

#### Speciation of iodine in water

Iodine was present both in inorganic and organic forms in all samples, but the species composition did not follow any obvious trend with location. However, inorganic species typically accounted for a larger percentage (mean = 69%) of total I in the majority of samples. This is comparable with data reported by Karim (2018) who measured I speciation in irrigation waters from Sulaimani province of Iraqi Kurdistan and found a higher proportion of inorganic species. A correlation (Pearson *r* = 0.490, *p* < 0.001) between DIC and inorganic I was observed which may reflect local geology. The smaller proportion of organic I (mean = 31%) may reflect the low concentration of DOC in water samples. The ratio of DOC (mg L^−1^) to organic I (µg L^−1^) (DOC/I_org_) was variable (range: 0.00–23,200, median: 21.4) across samples and did not show any significant trend with location.

Inorganic I in water samples was predominantly present as iodate (IO_3_^−^), possibly reflecting the alkaline conditions as nearly all samples had pH values above 7. Moran et al., (2002) reported that IO_3_^−^ is typically the predominant inorganic I species under alkaline conditions in water and soil. Our data are comparable with the findings reported by Gilfedder et al., (2009), Hansen (2011) and Karim (2018) that inorganic I is largely present as IO_3_^−^ in natural water. However, iodide (I^−^) dominated in a subset of samples potentially a consequence of its presence in this form in rocks and soil of the watershed. Smith and Butler (1979) reported that high concentrations of I^−^ in the Yarra river in Autralia were probably because of the dominant presence of I^−^ in soils and rocks in the river Yarra drainage basin, assuming no conversion of inorganic I species occurred in transit. An alternative reason for I^−^ enrichment in water may be the reduction in IO_3_^−^ to I^−^ due to microbial activity under reducing conditions (Li, Qian, et al., 2017).

### Selenium in water

#### Total selenium

Selenium in water samples ranged from 0.016 to 2.09 µg L^−1^ with a median concentration of 0.161 µg L^−1^ (supplementary information Table B1). A significant difference was observed in Se concentration between districts (*p* < 0.05). The results of this study are in agreement with other investigations. Wang et al., (1994) reported that the Se concentrations in river waters of several European countries, Japan and USA are largely < 1 µg L^−1^. Selenium concentrations in fresh water generally fall within the range 0.1–100 µg L^−1^ with most of the values below 3 µg L^−1^ (Fordyce, 2007, 2013). Watts and Mitchell (2009) reported an average concentration of 1.84 µg L^−1^ Se in surface water from a similar hilly area in Argentina. The low concentration of Se in the current study probably reflects the igneous and metamorphic geology of the area which is very low in Se. There was no correlation between concentration of Se, or individual Se species, and most water characteristics, such as pH and DOC (*p* > 0.05). However, Se concentration had a significant correlation (*r* = 0.549, *p* < 0.01) with DIC in water samples which may either reflect a calcareous origin for the Se or its pH-dependent solubility.

#### Speciation of selenium in water

Typically, Se was present both in inorganic and organic forms in water samples. On average, inorganic species accounted for the larger proportion (63%) of Se, while the remaining 37% was present in organic form. In all cases, the inorganic Se was present as selenate (Se^VI^); no selenite (Se^IV^) was detected in any of the water samples (supplementary information Table B1). The dominant presence of inorganic Se^VI^ is comparable with other studies. Wang et al., (1994) reported higher concentrations of inorganic Se, with Se^VI^ as the principal species, in river waters from Finland, Japan and USA. Other workers such as Conde and Alaejos (1997) and Cutter (1985) have also reported that Se^VI^ was the major inorganic species in river waters from various countries. Bujdoš et al., (2005) studied Se speciation in water from Slovakia which indicated Se^VI^ as the major species in water with pH > 7. The greater concentration of Se^VI^ may be explained by its greater solubility, and weaker adsorption on sediments, compared to Se^IV^ (Cary & Gissel-Nielsen, 1973; Fishbein, 1983; Wang et al., 1994; Wuilloud & Berton, 2014) or because Se is naturally present in this form in soils and rocks of the area.

### Soil characteristics

Details of basic soil characteristics including soil pH, texture, concentrations of inorganic and organic carbon and metal oxides are given in Table 1. Soils fell in the pH range of 6.70 to 8.33 (neutral to moderately alkaline). There was significant variation in pH across sampling districts (*p* < 0.05): the district Hunza-N had the highest pH value (8.07), while Astor had the lowest pH at 6.99. Particle size analysis demonstrated that soils in all districts were largely sandy loams apart from one site each in Hunza-N (N13) and Skardu (S24) where the soils were silty loams and medium loams, respectively (Table 1).

All the sampling districts had average soil inorganic carbon (SIC) contents of < 1%, but it varied significantly between sampling districts (*p* < 0.05). Districts Astor and Hunza-N accounted for the lowest and highest mean SIC contents of 0.001% and 0.726%, respectively. Some individual sites in Gilgit, Diamer and Astor did not have measurable SIC (Table 1) reflecting the highly diverse geology of the parent material. Soil organic carbon (SOC) across all sites was relatively low and ranged from 0.410 to 2.77% with a significant difference observed among districts (*p* < 0.05); Hunza-N had the highest SOC content of 2.34%. The other four districts, i.e. Gilgit, Diamer, Astor and Skardu, had mean values of 1.20%, 1.14%, 1.57% and 1.31% SOC, respectively. The sampling sites with relatively higher SOC had more numerous perennial fruit orchards, possibly reflecting inputs of plant residues and lack of ploughing which would tend to produce greater concentrations of soil humus. Combined metal oxides of Fe, Mn and Al showed a significant variation among districts (*p* < 0.05) and fell in the range of 2.22 to 7.75 g kg^−1^. Oxides of Mn and Al were less than 1.0 g kg^−1^ in all samples, and concentration of Fe oxides was 5.02 g kg^−1^ in Hunza-N samples; the remaining four districts, i.e. Gilgit, Diamer, Astor and Skardu, had average Fe oxide concentrations of 3.11, 2.86, 2.89 and 3.20 g kg^−1^, respectively.

### Iodine in soil

#### Total soil iodine

Concentrations of total soil iodine (I_T_) fell in the range of 273–1180 µg kg^−1^ with an average value of 685 µg kg^−1^ across all sites (Table 2). Average I_T_ concentrations in each district, i.e. Gilgit, Diamer, Hunza-N, Astor and Skardu were 674, 895, 650, 489 and 725 µg kg^−1^, respectively, and showed no significant regional differences (*p*> 0.05). Values of I_T_ were low compared to (i) the reported global mean value of 2600 µg kg^−1^ (ii) the average value (920 µg kg^−1^) reported for soils from other parts of Pakistan (Zia et al., 2014) (iii) values reported by Karim (2018) for the Kurdistan region of Iraq (4140 µg kg^−1^) and (iv) alluvium-derived soils worldwide (3560 µg kg^−1^) (Johnson, 2003); soils in Gilgit-Baltistan are largely alluvial in nature (Malik & Azam, 2009).

**Table 2:** Concentration of iodine in soil fractions and its extractability as a proportion of total soil iodine (I_T_)

District	Sample code	I_T_	I_sol_	I_ads_	I_sol_ extractability	I_ads_ extractability
		(µg kg^−1^)			(%)	
Gilgit	G01	796	17.5	20.3	2.20	2.54
	G02	784	14.3	19.5	1.83	2.48
	G03	391	9.93	8.87	2.54	2.27
	G04	454	9.44	12.6	2.08	2.78
	G05	944	14.3	18.8	1.52	2.00
Diamer	D06	813	21.9	19.5	2.70	2.40
	D07	1080	23.1	28.6	2.13	2.65
	D08	1027	24.7	29.0	2.40	2.83
	D09	1072	15.4	18.8	1.44	1.75
	D10	482	11.3	10.2	2.33	2.11
Hunza-N	N11	611	13.9	17.8	2.28	2.92
	H12	877	15.1	15.4	1.73	1.76
	N13	871	12.3	16.9	1.42	1.94
	H14	393	8.63	9.62	2.20	2.45
	H15	596	8.15	10.2	1.37	1.70
	N16	555	8.89	10.6	1.60	1.91
Astor	A17	445	7.32	10.3	1.64	2.32
	A18	492	8.38	12.2	1.70	2.48
	A19	273	7.39	10.5	2.71	3.83
	A20	679	10.1	14.6	1.49	2.15
	A21	558	6.66	11.8	1.19	2.11
Skardu	S22	742	15.9	17.9	2.14	2.42
	S23	1177	9.48	15.9	0.81	1.35
	S24	728	14.6	20.0	2.00	2.74
	S25	387	8.30	10.5	2.14	2.71
	S26	590	8.38	11.9	1.42	2.01

The majority of I in soils is generally derived from oceanic sources through atmospheric dry deposition and rainfall (Fuge & Johnson, 2015). Soil characteristics such as pH, texture and organic matter control retention and, over time, the concentration of total soil I (Bowley et al., 2019; Fuge & Johnson, 2015; Humphrey et al., 2020; Maity et al., 2017; Watts et al., 2019). Gilgit-Baltistan is far from the coast and located in the rain shadow area of the Himalayan mountains. Thus, the majority of soil I is believed to be geogenic, derived from the soil parent material. The igneous and metamorphic geology of the region (Malik & Azam, 2009) has less I than sedimentary rocks (Cox & Arai, 2014; Fuge & Johnson, 2015; Hou et al., 2009; Johnson, 2003). The input to soil I_T_ from irrigation water is likely to be minimal because of the low concentration of I in water sources. Furthermore, the soils are predominantly sandy with low organic carbon concentrations, which limits the ability of the soils to retain I (Johnson, 2003; Johnson et al., 2003; Köhler et al., 2019; Mohiuddin et al., 2019; Watts & Mitchell, 2009; Watts et al., 2019). The alkaline nature of the soils would also limit I retention; adsorption of I onto Fe and Al oxyhydroxides and rates of conversion to humus-bound I, are both lower under alkaline conditions (Johnson et al., 2003; Shetaya et al., 2012; Söderlund et al., 2017; Wang et al., 2019; Watts & Mitchell, 2009).

Principal component analysis (PC1 and PC2) revealed that various soil characteristics, especially those related to soil texture, accounted for 60% variation in soil I concentration (Fig. 2). No relationship was observed between the concentrations of metal oxides and I in all samples (*p* > 0.05); metal oxides are considered to be an important adsorption site for I in soil, but adsorption is most effective at low pH values (< 5) (Bowley et al., 2019; Humphrey et al., 2020; Schmitz & Aumann, 1994; Shetaya et al., 2012; Whitehead, 1973). Soil pH did not show a correlation with I_T_ in the majority of sampling districts except Skardu which showed a negative correlation (Pearson *r* = − 0.961, *p* = 0.009, *n* = 5) between soil pH and I_T_. The absence of correlation between soil pH and I_T_ may simply be due to the narrow range of pH of the soils, as suggested by Karim (2018) for soils in the Iraqi region of Kurdistan with a pH range similar to that of this study. However, it is contrary to the findings of Zia et al., (2014), Watts et al., (2015) and Bowley et al., (2019) who reported a negative correlation in soils of Pakistan, Malawi and Northern Ireland, respectively. A positive correlation (*r* = 0.900, *p* = 0.037) between SOC and I_T_ was only observed in Skardu. Soil humus is important for retaining soil I; however, the narrow range of SOC (< 3%) in all samples may have masked any underlying causal relationship. Fuge and Johnson (1986), Johnson et al., (2002), Zia et al., (2014), Watts et al., (2015) and Bowley et al., (2019) all reported a positive correlation between the concentrations of soil organic matter and I in samples from the UK, Morocco, Pakistan, Malawi and Northern Ireland, respectively. However, Karim (2018) reported no correlation with SOC in soils from Iraqi Kurdistan.

#### Fractionation of soil iodine

##### Soluble iodine

The soluble fraction of I (I_sol_, extracted with 0.01 M KNO_3_) accounted for ≤ 3% of I_T_ in all samples (Table 2). It ranged from 6.66 to 24.7 µg kg^−1^ with an overall median of 10.7 µg kg^−1^. While the concentration of I_sol_ varied between samples, the proportion of I_T_ that was extractable in 0.01 M KNO_3_ was almost the same in each district and did not reveal significant differences between districts (*p* > 0.05). The small percentage of I_T_ available as I_sol_ (Table 2) is comparable with other studies. It was reported that that only 1–12% of I was water-soluble in soil samples from Dagestan, USSR (Johnson, 2003). Fuge and Johnson (1986) reported that less than 10% of I_T_ was extractable with water in approximately 80% of soils mainly from Wales (*n* = 183). Soils from other parts of Pakistan (Zia et al., 2014) and other countries such as Ukraine (Duborska et al., 2020; Hou et al., 2009), Sweden (Hou et al., 2009), Denmark (Hansen et al., 2011), Malawi (Watts et al., 2015), Kurdistan (Karim, 2018) and Slovakia (Duborska et al., 2020) have revealed similar average proportions of water-soluble I concentrations: 2.36%, 12.7%, 3%, 4.8%, 1.38%, 1.59% and 4.4% of total soil I, respectively. Small proportions of water soluble I have also been measured for German soils (< 4% of I_T_) (Hou et al., 2009; Humphrey et al., 2018; Schmitz & Aumann, 1994).

**Fig. 3:**
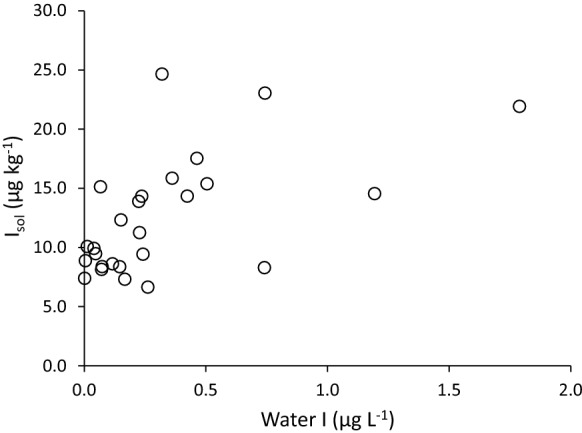
Relationship between iodine in irrigation water and soluble soil iodine (I_sol_) in all samples as one dataset

Soil characteristics including pH and SOC did not show a relationship with I_sol_. It is possible that variation in I_sol_ concentration across different districts may be due to the variation in I concentration in irrigation water. The concentration of I in irrigation water showed a positive correlation with I_sol_ (*r* = 0.599, *p* = 0.001) when samples of all districts were considered as one data set (Fig. 3).

##### Adsorbed iodine

The adsorbed fraction of soil iodine (I_ads_, extracted with 0.016 M KH_2_PO_4_) ranged from 8.87 to 29.0 µg kg^−1^ and had a median concentration of 15.0 µg kg^−1^. It comprised < 4.0% of I_T_ on average for all samples. Shetaya et al., (2012) and Karim (2018) reported a slightly higher ratio of 1–9% and 10.7% present as I_ads_ in their fractionation experiments on soils from the UK and Kurdistan region of Iraq, respectively. As a percentage of I_T_, I_ads_ varied across the sampling districts but showed no significant correlation with soil properties (*p* > 0.05), probably because of similar soil properties that might affect I adsorption (SOC, oxide content, clay content and pH) (Bowley et al., 2019; Duborska et al., 2020; Humphrey et al., 2018, 2020; Medrano-Macías et al., 2016).

The kd value for I (Eq. 1) was very low (1.07 ± 0.274), probably due to coarse texture and low organic carbon contents, suggesting very limited buffering of available soil I against leaching losses and plant uptake.

#### Speciation of soil iodine

Iodine speciation was carried out on the soluble and adsorbed fractions. In all of the districts most of the I in both I_sol_ and I_ads_ fractions was present as *organic I* (Tables 3 and 4). The median concentrations of organic I in the soluble and adsorbed fractions were 10.4 and 13.8 µg kg^−1^ which accounted for 98% and 90% of the I_sol_ and I_ads_, respectively, across all samples. The large proportion of organic I is comparable with findings from other studies. Hu et al., (2007) reported that a large proportion of I (> 90%) in soils is present bound to humic and fulvic acids in samples from USA. Bowley et al., (2016, 2019) and Humphrey et al., (2020) also reported higher concentration of organic I compared to inorganic I in soils from the UK. The ratio of inorganic species, iodide (I^−^) and iodate (IO_3_^−^), was variable across the samples and inconsistent within different districts (supplementary information Table B2 and B3). However, on average, I^−^ generally accounted for a larger proportion of inorganic I in both I_sol_ (63%) and I_ads_ (84%) fractions across all districts; this is comparable to other studies such as Yamada et al., (1999), Hu et al., (2005) and Hu etal., (2007). Iodate is sorbed more strongly in most soils than I^−^ and is therefore less easily extracted (Fuge & Johnson, 2015; Hu et al., 2005, 2007; Humphrey et al., 2020). The other reason for a larger I^−^ presence is probably its stability in the soil solution. Iodide is the dominant inorganic species in most soil solutions because of its stability over a wide range of Eh and pH values (Söderlund et al., 2011; Cox & Arai, 2014).

**Table 3:** Concentration of selenium in soil fractions and its extractability as a proportion of total soil selenium (Se_T_)

District	Sample code	Se_T_	Se_sol_	Se_ads_	Se_TMAH_	Se_sol_	Se_ads_	Se_TMAH_
		(µg kg^−1^)				(% Se_T_)		
Gilgit	G01	168	2.12	3.09	83.6	1.26	1.83	49.6
	G02	165	2.00	3.28	92.0	1.21	1.99	55.7
	G03	289	4.45	4.26	139	1.54	1.47	48.0
	G04	123	1.65	2.25	51.4	1.34	1.83	41.8
	G05	202	2.78	4.09	149	1.38	2.03	73.9
Diamer	D06	109	2.11	2.25	54.3	1.93	2.06	49.7
	D07	199	3.41	4.61	106	1.72	2.32	53.1
	D08	143	1.79	1.91	59.7	1.25	1.34	41.9
	D09	120	1.33	1.26	70.7	1.12	1.05	59.2
	D10	92.7	1.07	1.00	32.4	1.15	1.08	34.9
Hunza-N	N11	430	5.11	7.41	177	1.19	1.72	41.1
	H12	329	2.08	1.99	106	0.631	0.604	32.2
	N13	453	6.52	7.82	293	1.44	1.73	64.6
	H14	333	4.65	4.77	165	1.40	1.44	49.5
	H15	265	2.20	1.62	108	0.830	0.613	40.6
	N16	265	2.52	1.98	126	0.951	0.748	47.5
Astor	A17	151	1.57	1.13	66.8	1.04	0.749	44.2
	A18	135	1.64	1.47	65.2	1.22	1.09	48.4
	A19	150	1.42	1.70	57.0	0.948	1.14	38.1
	A20	172	1.22	1.26	61.7	0.708	0.729	35.8
	A21	109	0.951	0.987	47.8	0.87	0.907	43.9
Skardu	S22	178	3.67	2.21	91.3	2.06	1.24	51.3
	S23	219	1.87	2.01	146	0.852	0.919	66.7
	S24	279	3.82	5.21	128	1.37	1.87	46.1
	S25	216	2.82	2.51	82.6	1.31	1.17	38.3
	S26	151	1.40	0.944	45.5	0.927	0.626	30.2

### Selenium in soil

#### Total soil selenium

The average total soil Se concentration (Se_T_) across all districts was 209 µg kg^−1^ and ranged from 92.7 to 453 µg kg^−1^ (Table 3). Hunza-N district had the highest mean Se_T_ of 346 µg kg^−1^ and was significantly different from the other four districts (*p* < 0.05). The districts of Gilgit, Diamer, Astor and Skardu had mean Se_T_ concentrations of 190, 132, 143 and 208 µg kg^−1^, respectively, which were not significantly different from each other (*p* > 0.05). All sites had Se_T_ concentrations less than the global mean of 400 µg kg^−1^ (Fordyce et al., 2000; Xing et al., 2015) except two locations in Hunza-N (N11 and N13) (Table 3). The generally low Se_T_ concentrations in the area probably reflect the geology of the area, the sandy soil texture and low organic carbon concentrations. The geology of the study area is dominated by metamorphic and igneous rocks, which usually contain less Se compared to sedimentary rocks (Alexander, 2015; Fordyce et al., 2010, 2013; Koljonen, 1973). Underlying rock type has a major role in Se concentration in most soils (Fordyce, 2007, 2013; Fordyce et al., 2009). Principal components analysis revealed that a sandy soil texture was found to be negatively correlated with Se_T_ concentration (Fig. 2). Sandy soils generally retain less Se compared to clayey soils (Antanaitis et al., 2008; Lopes et al., 2017), and organic carbon plays an important role in retaining soil Se (Gustafsson et al., 1993; Jones et al., 2017; Li, Liang, et al., 2017; Lopes et al., 2017; Supriatin et al., 2016; Xing et al., 2015). The slightly greater Se_T_ in two sites (N11 and N13) may be a localised effect possibly reflecting long-term use of irrigation water; the corresponding irrigation waters of N11 and N13 had relatively high Se concentrations.

**Fig. 4:**
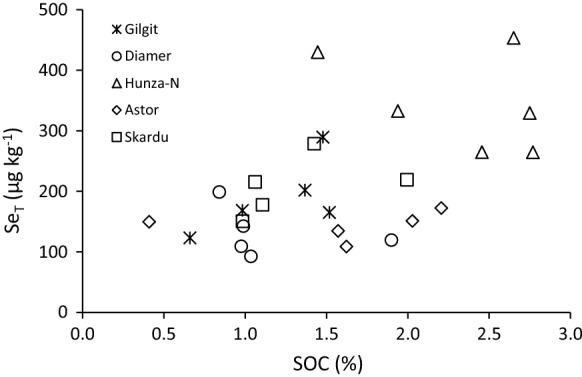
Correlation between concentrations of soil organic carbon (SOC) and total soil selenium (Se_T_)

Moreover, soils from these sites had relatively high organic carbon contents compared to other sites. In most cases, the contribution to Se_T_ in soils from seasonal irrigation is likely to be low because of the generally low Se_w_ concentrations in irrigation water and the predominance of soluble Se^VI^ in water. Soil organic carbon had a positive correlation (*r* = 0.509, *p* < 0.005) with Se_T_ for all data considered together, but there was no correlation for intra-district data (Fig. 4). This could be the result of small sample sizes and a narrow range of %SOC in each district. The SIC also showed a positive correlation (*r* = 0.668, *p* < 0.001) with Se_T_ and with the soil Se fractions (soluble, adsorbed and humus-bound) for the whole set of data but again there were no significant correlations for the intra-district data.

The majority of the soils in this study were marginally deficient in Se based on the threshold values for Se deficiency (< 125 µg kg^−1^) and marginal deficiency (> 125—175 µg kg^−1^) in soils reported by Fordyce et al., (2009). The typically low Se_T_ concentrations in the area are in the range for sandy soils reported in other parts of the world. For example, sandy soils in Poland, Lithuania, Russia, Finland and Canada had 140, 140, 180, 210 and 270 µg kg^−1^ of Se_T_, respectively (Kabata-Pendias & Mukherjee, 2007). Watts et al., (2010) reported 300 µg kg^−1^ Se in a mountainous area of San Juan in Argentina which is in a similar range to this study. Similarly, Chilimba et al., (2011) reported an average Se concentration of 194 µg kg^−1^ in Malawian soils which is typical of the region due to its geology and advanced weathering of many landscapes.

#### Fractionation of soil selenium

##### Soluble selenium

The soluble Se fraction (Se_sol_, extracted with 0.01 M KNO_3_) ranged from 0.95 to 6.52 µg kg^−1^ with mean and median values of 2.54 and 2.09 µg kg^−1^. A significant variation in Se_sol_ was seen between districts (*p* < 0.05). The districts of Hunza-N and Astor had the highest (3.84 µg kg^−1^) and lowest (1.36 µg kg^−1^) values of Se_sol_, respectively. Districts Skardu, Gilgit and Diamer had mean Se_sol_ values of 2.71, 2.60 and 1.94 µg kg^−1^, respectively. The concentration of Se_sol_ as a percentage of soil Se_T_ (%Se_sol_) was very low and typically accounted for < 2% of Se_T_ across all sites (Table 3). There was no significant variation between districts (*p* > 0.05) which also suggests %Se_sol_ was independent of soil Se_T_. The low extractability of Se_sol_ in soils is comparable with other studies. Karim (2018) used the same sequential extraction procedure and found that %Se_sol_ ranged from 0.096 to 2.18% in Kurdistan soils. Wang et al., (2012) reported < 1% of soluble Se in agriculture soils of Shaanxi province in China. Tan etal., (2002) and Xing et al., (2015) studied the concentration of water soluble Se in different soil types in China and found it varied from 1.07–6.69% and 0.28–1.45%, respectively. The use of a parallel single extraction method on soils from the UK showed that water-soluble Se accounted for 1.4—14% of the total soil Se (Tolu et al., 2011). Keskinen et al., (2009) studied the fate of residual Se in Finland soils, amended with Se fertilizers and observed that soluble Se account for approximately 1% of Se_T_. Similarly, Ligowe et al., (2020) investigated the fate of residual isotopically labelled ^77^Se fertilizer in Malawian soils, using the same sequential extraction procedure as used in the current study and found that the soluble fraction of Se accounted for ~ 3% of total ^77^Se applied in the preceding year.

##### Adsorbed selenium

Adsorbed Se (Se_ads_) may represent the Se fraction associated with metal oxides. The range of Se_ads_ concentrations (0.944–7.82 µg kg^−1^) was similar to that of Se_sol_ (Table 3); the average Se_ads_ in all samples was 2.81 µg kg^−1^. A significant variation in Se_ads_ concentration among districts was observed with Hunza-N exhibiting the highest mean value of 4.27 µg kg^−1^. The average concentrations in other districts (Gilgit, Skardu, Diamer and Astor) were 3.39, 2.58, 2.20 and 1.31 µg kg^−1^, respectively. Adsorbed Se as a percentage of soil Se_T_ (%Se_ads_) was not significantly different from that of Se_sol_ (*p*> 0.05). For all samples, Se_ads_ recovery ranged from 0.604 to 2.32% and had a mean value of 1.31%. The average values of %Se_ads_ for Gilgit, Diamer, Skardu, Hunza-N and Astor were 1.83, 1.57, 1.17, 1.14 and 0.923%, respectively. The low recovery of Se_ads_ is comparable with other investigations. Ligowe et al., (2020) reported an average Se_ads_ recovery of < 3% in Malawian soils. Karim (2018) reported a comparable range of %Se_ads_ with a mean value of 1.88% in 97 soil samples from Kurdistan. Mathers (2015) determined %Se_ads_ for 78 Malawi and 236 UK soils and reported values of 3.12% and 2.62%, respectively. The results of a Malawi national survey, including 87 soil samples, reported %Se_ads_ values of < 1—8% (Chilimba et al., 2011); Stroud et al., (2012) reported %Se_ads_ values of 1.1–3.4% for UK soils. Some studies have also reported higher levels of %Se_ads_: Tolu et al., (2011) measured 20% in a clay loam soil from the UK; Schilling et al., (2011) and Schilling etal., (2014) reported 12—35% and 12—27% in German and Indian soils, respectively; Keskinen et al., (2009) found 15—20% in Finnish soils.

##### Humus-bound selenium

Concentrations of humus-bound Se (Se_TMAH_) ranged from 32.4 to 293 µg kg^−1^ with an average value of 100 µg kg^−1^ considering all samples together (Table 3). There was a significant variation between districts; concentrations in Hunza-N, Gilgit, Skardu, Diamer and Astor were 162, 103, 98.9, 59.7 and 64.5 µg kg^−1^, respectively. The extractability of Se_TMAH_, as a percentage of Se_T_, ranged from 30 to 74% with an overall average of 47%; there was no significant variation between districts (*p* > 0.05). For the majority of samples (65%), Se_TMAH_ was less than 50% of Se_T_. This suggests that a substantial amount of Se is present in a refractory pool, resistant to dissolution in TMAH and extractable only with the HF-HClO_4_-HNO_3_ digestion procedure. This form of Se is likely to be present within mineral structures (Mathers, 2015). Comparable (average) recovery of Se_T_ (41%) with TMAH was observed in 97 soil samples from Kurdistan (Karim, 2018). A wide range of Se recoveries in soils and sediments using alkaline extractions has been reported, including 50% (Séby et al., 1997), 29–37% (Ponce de León et al., 2003), 35–50% (Keskinen et al., 2009), 31.8–52% (Qin et al., 2012), 31.9–70.1% (Schilling et al., 2014), in soils and sediments from Ireland, Canada, Finland, China and India, respectively.

### Speciation of soil selenium

Speciation analysis was performed on both components of ‘available Se’: Se_sol_ and Se_ads_ extracts (supplementary information Table B4 and B5). A large proportion of Se_sol_ was present as organic-Se with an average value of 66.9% and a range from 43.5% to 89.9% considering all samples together. The average proportion of organic Se_sol_ did not show significant variation between districts (*p* > 0.05) and consisted of 60.1, 65.8, 69, 75.5 and 64% of total Se_sol_ in Gilgit, Diamer, Hunza-N, Astor and Skardu, respectively. The soluble organic Se is probably linked to soil humus acids, but soluble organic Se may also be present in parent materials. Kulp and Pratt (2004) reported that a large proportion of soluble Se was present as organic Se in different rocks from the USA. Similarly, Zhang and Moore (1996) reported a large proportion of the soluble Se fraction in wetland sediments from Montana was organically bound.

In the adsorbed fraction (Se_ads_), there was a wide variation in speciation with the % organic Se ranging from 0 to 87% with a mean value of 39.7% for the whole data set. There was a significant difference between districts (*p* < 0.05) with Astor and Skardu accounting for the highest (68.4%) and lowest (19.2%), respectively. The proportions of organic Se_ads_ in other districts were: 30.6% (Hunza-N), 32.7% (Gilgit) and 49.5% (Diamer). The variation observed in adsorbed organic species is comparable with the results described in Stroud et al., (2010) who reported a range of 30 – 87% organic Se in phosphate extracts of soils from different parts of the UK. Similarly, Kulp and Pratt (2004) reported a range of 13.6–85% organic-Se in phosphate extractions of parent rocks.

The inorganic Se in Se_sol_ and Se_ads_ was present as both Se^IV^ and Se^VI^, but the proportion of inorganic Se present as Se^IV^ in the soluble and adsorbed fractions ranged from 83.7–100% to 94.1–99.9%, respectively (supplementary information Table B4 and B5); there was no difference between districts (*p* > 0.05). The large proportion of Se^IV^ in the soluble inorganic fraction contradicts other investigations in the literature, but for the adsorbed fraction the values were comparable to other studies. Karim (2018) and Wang et al., (2012) reported that inorganic Se_sol_ was largely present as Se^VI^ in Se_sol_ fraction in soils from Kurdistan and China; similarly, Kulp and Pratt (2004) also reported a large proportion of Se^VI^ in inorganic Se_sol_. The large proportion of Se^IV^ in inorganic Se_sol_ in the current study could be due to its presence in the geology of the area. By contrast, the large proportion of Se^IV^ in inorganic Se_ads_ was consistent with other investigations. Karim (2018) found that 96% of inorganic Se_ads_ was present as Se^IV^. Similarly, a study of Se speciation and extractability in Dutch agricultural soils found that Se was largely present as Se^IV^ in the adsorbed fraction extracted with ammonium oxalate (Supriatin et al., 2016). Wang et al., (2012) and Stroud et al., (2010) observed that Se^IV^ was the only inorganic species detected in an adsorbed fraction of soil samples from Shaanxi province in China and different parts of the UK, respectively; Kulp and Pratt (2004) reported the presence of only Se^IV^ in their adsorbed fraction.

Selenite is strongly adsorbed on soil surfaces compared to Se^VI^, but the average kd value for Se^IV^ (Eq. 1) was very low (1.27 ± 0.214). As suggested for I, this was probably due to the coarse texture and low humus content of the soil.

The low kd value reflects the lack of a substantial buffer mechanism for available Se. Not only is the soluble Se_sol_ very low, and mainly organic, but the ability of the soil to replenish Se in solution from Se_ads_ following depletion by leaching or plant uptake is also very poor. Taking all the factors above into account, it is clear that the Se status of Gilgit-Baltistan region is exceptionally low.

## Conclusions

**Fig. 5:**
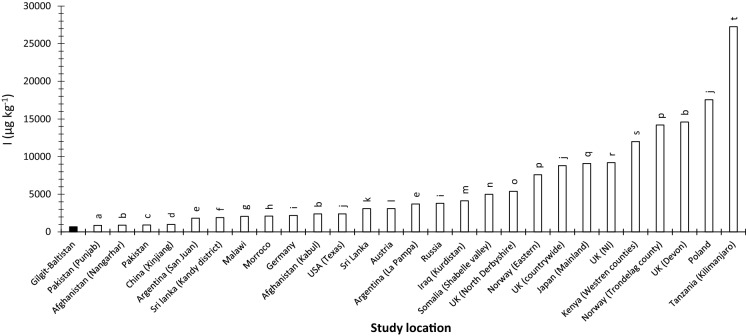
Comparison of mean Gilgit-Baltistan soil I_T_ with average soil I concentration from other studies worldwide as well as in Pakistan. The dark colour bar represents I concentration in Gilgit-Baltistan soil. *Sources* ^a^Zia et al., 2017; ^b^Watts & Mitchell, 2009; ^c^Zia et al., 2014; ^d^Fordyce et al., 2003; ^e^Watts et al., 2010; ^f^Dissanayake & Chandrajith, 1996; ^g^Watts et al., 2015; ^h^Johnson et al., 2002; ^i^Ashworth, 2009; ^j^Johnson, 2003; ^k^Fordyce et al., 2000; ^l^Gerzabek et al., 1999; ^m^Karim, 2018; ^n^Ali, 2020; ^o^Fuge & Long, 1989; ^p^Låg & Steinnes, 1976; ^q^Yamasaki et al., 2015; ^r^Bowley, 2013; ^t^Watts et al., 2019

**Fig. 6:**
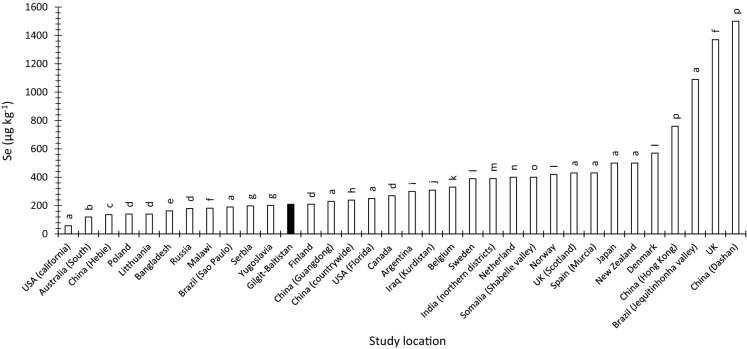
Comparison of mean Gilgit-Baltistan soil Se_T_ concentration with average soil Se concentration from other studies worldwide. The dark colour bar represents Se concentration in Gilgit-Baltistan soil. *Sources* ^*a*^Lopes et al., 2017*; *^*b*^Ramkissoon 2020; ^*c*^Fordyce et al., 2003*; *^*d*^Kabata-Pendias & Mukherjee, 2007*; *^*e*^Rahman et al., 2013*; *^*f*^Mathers, 2015*; *^*g*^Maksimovic et al., 1992*; *^*h*^Tan et al., 2002*; *^*i*^Watts et al., 2010*; *^*j*^Karim, 2018*; *^*k*^De Temmerman et al., 2014*; *^*l*^Girling, 1984*; *^*m*^Yadav et al., 2005*; *^*n*^Supriatin et al., 2016*; *^*o*^Ali, 2020*; *^*p*^Xing et al., 2015

The average concentrations of I_T_ and Se_T_ in Gilgit-Baltistan soils were 685 and 209 µg kg^−1^, respectively, which are lower than the global average of soil I_T_ (2600 µg kg^−1^) and Se_T_ (400 µg kg^−1^), and most of the reported values for I_T_ and Se_T_ in other parts of the world (Figs. 5 and 6). The concentration of I and Se in soil parent materials (igneous and metamorphic rocks) is low, and the contribution from other sources (marine and rainfall) is likely to be negligible because Gilgit-Baltistan is about 1400 km away from the nearest sea and is located in a rain shadow region with minimum rainfall. Soils in the area have a coarse texture, low organic carbon and high pH which restricts their ability to retain I and Se. The input to soil I_T_ and Se_T_ from irrigation water is likely to be minimal because of the low concentrations of I (0.01–1.79 µg L^−1^) and Se (0.016–2.09 µg L^−1^) in irrigation water. The soluble and adsorbed fractions of soil I and Se, which are considered to be available for plant uptake, accounted for < 7% and < 3% of total soil I and Se content, respectively. The distribution coefficient (kd) for I (1.07 ± 0.274) and Se (1.27 ± 0.214) was very low suggesting very limited buffering of available I against leaching losses and plant uptake. Thus, not only are the I_sol_ and Se_Sol_ concentrations very low but the ability of the soil to replenish I and Se in solution from I_ads_ and Se_ads_ following depletion by leaching or plant uptake is also very poor. Furthermore, I and Se in the soluble and adsorbed fractions was predominantly present as organic species which may not be available to plants. All these factors demonstrate that the low status of I and Se in the Gilgit-Baltistan environment is the product of several co-existing factors.

The low concentration of I and Se in Gilgit-Baltistan soil and water may be reflected in locally grown crops and ultimately in the local population because the population in the area largely consumes locally grown agricultural produce which restricts their access to dietary I and Se form other sources.

## Supplementary Information

Below is the link to the electronic supplementary material.

Supplementary file1 (DOCX 606 kb)

## Data Availability

The authors confirm that the summary of data supporting the findings of this study is available within the article. Detailed data are available from the corresponding author upon request.
