# Supplementary material for: Multiple geochemical factors may cause iodine and selenium deficiency in Gilgit-Baltistan, Pakistan
Source: Environ Geochem Health. 2021 Apr 24;43(11):4493–513. doi: 10.1007/s10653-021-00936-9 (PMC8528784; doi:10.1007/s10653-021-00936-9)
Supplement: Supplementary file 1 — Supplementary file1 (DOCX 606 kb) [file 10653_2021_936_MOESM1_ESM.docx]

# Supplementary information

**Multiple geochemical factors may cause iodine and selenium deficiency in Gilgit Baltistan, Pakistan**

Saeed Ahmad^1^, Elizabeth H. Bailey^1*^, Muhammad Arshad^2^, Sher Ahmed^2^, Michael J. Watts^3^, Scott D. Young^1^

^1^Division of Agricultural and Environmental Sciences, School of Biosciences, University of Nottingham, Sutton Bonington Campus, Loughborough, Leicestershire LE12 5RD, United Kingdom

^2^Mountain Agriculture Research Centre Gilgit (Pakistan Agricultural Research Council), Gilgit-Baltistan, Pakistan

^3^Centre for Environmental Geochemistry, Inorganic Geochemistry, British Geological Survey, Nottingham NG12 5GG, United Kingdom

*Corresponding author: [liz.bailey@nottingham.ac.uk](mailto:liz.bailey@nottingham.ac.uk)


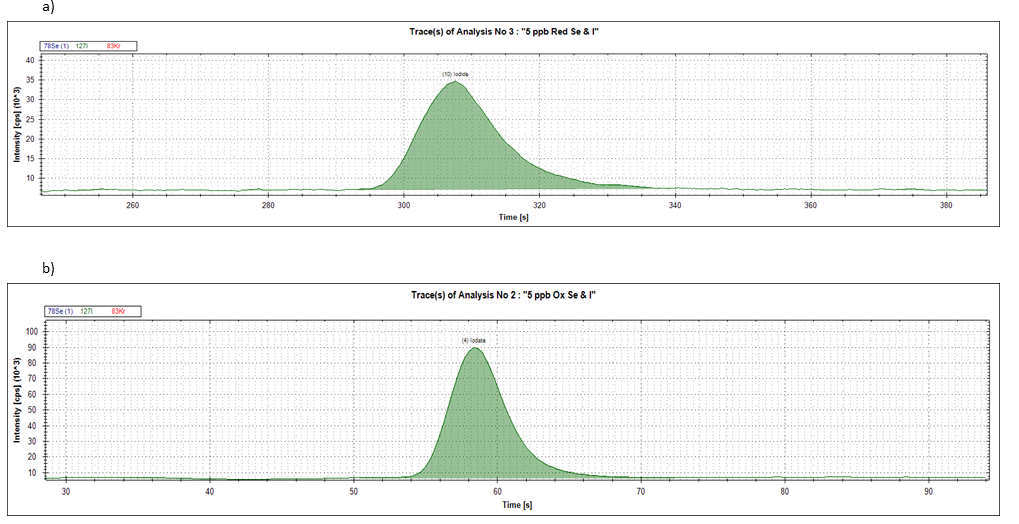


Fig. A1. Chromatographs of reduced and oxidised standards (5 µg L^-1^) of iodine - a) iodide (I^-^) and b) iodate (IO_3_^-^)


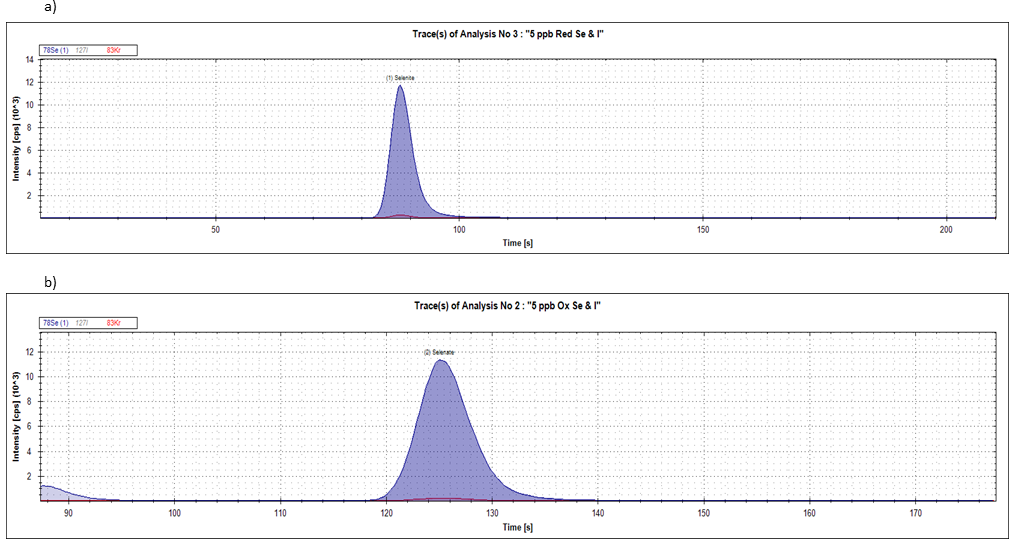


Fig. A2. Chromatographs of reduced and oxidised standards of selenium (5 µg L^-1^) - a) selenite (Se^IV^) and b) selenate (Se^VI^)

Table B1. Basic characteristics, iodine and selenium concentrations and their speciation in water samples. The initial in column “Sample number” represents district. “G-Gilgit, D-Diamer, HN-Hunza-N, A-Astor and S-Skardu”.

| **Sample number** | **pH** | **EC** | **DOC** | **DIC** | **CaCO_3_** | **Iodine** | **Organic iodine** | **inorganic iodine** | **Iodate** | **Iodide** | **Selenium** | **Organic selenium** | **inorganic selenium** | **Selenate** | **Selenite** |
| --- | --- | --- | --- | --- | --- | --- | --- | --- | --- | --- | --- | --- | --- | --- | --- |
|  |  | **(µS cm^-1^)** | **(mg L^-1^)** | | | **(µg/L)** | | | | | | | | | |
| G-01 | 8.86 | 96.0 | 0.487 | 7.41 | 61.7 | 0.463 | 0.250 | 0.213 | 0.213 | BDL | 0.088 | 0.036 | 0.050 | 0.052 | BDL |
| G-02 | 7.52 | 86.0 | 0.89 | 6.70 | 55.8 | 0.236 | 0.017 | 0.219 | 0.219 | BDL | 0.067 | 0.031 | 0.040 | 0.035 | BDL |
| G-04 | 8.25 | 187 | NA | 17.3 | 144 | 0.029 | 0.017 | 0.012 | 0.012 | BDL | 0.143 | 0.049 | 0.090 | 0.094 | BDL |
| G-05 | 8.22 | 157 | 1.89 | 15.3 | 127 | 0.230 | 0.016 | 0.214 | 0.214 | BDL | 0.209 | 0.064 | 0.140 | 0.145 | BDL |
| G-06 | 8.12 | 202 | 0.025 | 13.8 | 115 | 0.040 | 0.012 | 0.028 | 0.028 | BDL | 0.339 | 0.089 | 0.250 | 0.250 | BDL |
| G-07 | 7.63 | 118 | 1.27 | 4.22 | 35.2 | 0.242 | 0.037 | 0.204 | 0.204 | BDL | 0.073 | 0.033 | 0.040 | 0.040 | BDL |
| G-09 | 7.56 | 94.0 | 1.08 | 5.24 | 43.6 | 0.286 | 0.075 | 0.211 | 0.211 | BDL | 0.073 | 0.037 | 0.040 | 0.036 | BDL |
| G-11 | 7.87 | 95.0 | 1.38 | 5.49 | 45.8 | 0.391 | 0.178 | 0.213 | 0.213 | BDL | 0.104 | 0.040 | 0.060 | 0.064 | BDL |
| G-14 | 8.08 | 128 | 1.19 | 8.01 | 66.7 | 0.424 | 0.223 | 0.200 | 0.200 | BDL | 0.161 | 0.044 | 0.120 | 0.117 | BDL |
| G-15 | 7.68 | 93.0 | 1.61 | 5.95 | 49.6 | 0.220 | 0.024 | 0.197 | 0.197 | BDL | 0.109 | 0.044 | 0.060 | 0.064 | BDL |
| G-17 | 7.89 | 110 | 1.45 | 6.69 | 55.8 | 0.225 | 0.032 | 0.193 | 0.193 | BDL | 0.123 | 0.054 | 0.070 | 0.070 | BDL |
| G-18 | 7.84 | 106 | 1.07 | 5.14 | 42.9 | 0.200 | 0.006 | 0.194 | 0.194 | BDL | 0.041 | 0.016 | 0.020 | 0.025 | BDL |
| G-20 | 8.19 | 353 | 1.67 | 13.4 | 112 | 0.360 | 0.122 | 0.238 | 0.188 | 0.050 | 1.15 | 0.299 | 0.850 | 0.849 | BDL |
| G-21 | 8.11 | 184 | 0.614 | 13.0 | 109 | 0.132 | 0.106 | 0.026 | 0.026 | BDL | 0.461 | 0.120 | 0.340 | 0.341 | BDL |
| G-22 | 7.76 | 240 | 0.957 | 5.14 | 42.9 | 0.119 | 0.088 | 0.031 | 0.031 | BDL | 0.204 | 0.061 | 0.140 | 0.143 | BDL |
| G-23 | 7.89 | 377 | 0.921 | 17.0 | 142 | 0.029 | NA | 0.029 | 0.029 | BDL | 2.09 | 0.581 | 1.51 | 1.51 | BDL |
| D-31 | 8.23 | 297 | 3.66 | 12.5 | 104 | 0.280 | 0.093 | 0.186 | 0.165 | 0.021 | 0.437 | 0.156 | 0.280 | 0.281 | BDL |
| D-33 | 8.23 | 242 | 0.634 | 10.3 | 86.2 | 0.012 | 0.006 | 0.007 | 0.001 | 0.005 | 0.320 | 0.123 | 0.200 | 0.197 | BDL |
| D-35 | 8.43 | 220 | 3.51 | 17.0 | 142 | 1.79 | 1.60 | 0.187 | 0.171 | 0.017 | 0.109 | 0.079 | 0.030 | 0.030 | BDL |
| D-36 | 8.07 | 94.0 | 0.982 | 4.65 | 38.7 | 0.124 | 0.023 | 0.101 | 0.020 | 0.081 | 0.097 | 0.027 | 0.070 | 0.070 | BDL |
| D-37 | 8.32 | 248 | 0.902 | 9.72 | 81.0 | 0.743 | 0.334 | 0.409 | 0.397 | 0.012 | 0.160 | 0.058 | 0.100 | 0.103 | BDL |
| D-39 | 7.93 | 156 | 1.17 | 9.28 | 77.3 | 0.048 | 0.014 | 0.034 | 0.034 | BDL | 0.149 | 0.047 | 0.100 | 0.101 | BDL |
| D-40 | 7.86 | 63.0 | 1.78 | 4.02 | 33.5 | 0.572 | 0.417 | 0.156 | 0.156 | BDL | 0.062 | 0.041 | 0.020 | 0.021 | BDL |
| D-41 | 7.84 | 77.0 | 1.55 | 3.02 | 25.2 | 0.227 | 0.067 | 0.160 | 0.160 | BDL | 0.016 | 0.016 | NA | BDL | BDL |
| D-42 | 8.17 | 192 | 0.554 | 14.3 | 119 | 0.241 | 0.059 | 0.181 | 0.159 | 0.022 | 0.381 | 0.145 | 0.240 | 0.236 | BDL |
| D-45 | 8.43 | 87.0 | 1.37 | 5.60 | 46.6 | 0.830 | 0.653 | 0.176 | 0.157 | 0.020 | 0.068 | 0.054 | 0.010 | 0.014 | BDL |
| D-46 | 8.17 | 129 | 1.48 | 10.0 | 83.7 | 0.252 | 0.075 | 0.178 | 0.155 | 0.022 | 0.097 | 0.051 | 0.050 | 0.046 | BDL |
| D-48 | 8.26 | 140 | 1.08 | 10.7 | 89.2 | 0.320 | 0.163 | 0.157 | 0.157 | BDL | 0.057 | 0.048 | 0.010 | 0.009 | BDL |
| D-51 | 8.14 | 105 | 0.960 | 9.14 | 76.2 | 0.505 | 0.331 | 0.173 | 0.158 | 0.015 | 0.096 | 0.062 | 0.030 | 0.034 | BDL |
| HN-52 | 8.34 | 211 | 0.955 | 17.3 | 144 | 0.224 | 0.048 | 0.176 | 0.156 | 0.020 | 0.590 | 0.174 | 0.420 | 0.416 | BDL |
| HN-54 | 8.04 | 239 | 0.430 | 11.9 | 98.7 | 0.067 | NA | 0.067 | 0.018 | 0.050 | 0.444 | 0.126 | 0.320 | 0.318 | BDL |
| HN-55 | 8.15 | 246 | 0.49 | 3.92 | 32.7 | 0.019 | NA | 0.019 | 0.019 | BDL | 0.026 | 0.012 | 0.010 | 0.014 | BDL |
| HN-56 | 6.99 | 63.0 | 0.720 | 5.36 | 44.7 | 0.110 | 0.090 | 0.021 | 0.021 | BDL | 0.039 | 0.014 | 0.030 | 0.025 | BDL |
| HN-57 | 8.28 | 245 | 2.94 | 26.4 | 220 | 0.151 | 0.108 | 0.043 | 0.043 | BDL | 0.584 | 0.152 | 0.430 | 0.432 | BDL |
| HN-59 | 8.22 | 383 | 1.37 | 17.3 | 144 | 0.116 | 0.046 | 0.070 | 0.038 | 0.031 | 0.455 | 0.165 | 0.290 | 0.290 | BDL |
| HN-61 | 7.89 | 112 | 1.09 | 3.06 | 25.5 | 0.071 | 0.018 | 0.053 | 0.027 | 0.026 | 0.104 | 0.030 | 0.070 | 0.074 | BDL |
| HN-62 | 7.91 | 143 | NA | 11.0 | 91.6 | 0.010 | 0.001 | 0.009 | 0.009 | 0.001 | 0.248 | 0.059 | 0.190 | 0.189 | BDL |
| HN-64 | 7.92 | 164 | 1.43 | 11.4 | 95.0 | 0.214 | 0.042 | 0.172 | 0.029 | 0.142 | 0.187 | 0.065 | 0.120 | 0.122 | BDL |
| HN-65 | 7.90 | 138 | 1.86 | 9.21 | 76.8 | 0.145 | 0.020 | 0.125 | 0.028 | 0.097 | 0.261 | 0.059 | 0.200 | 0.202 | BDL |
| HN-66 | 8.01 | 183 | 2.50 | 13.7 | 114 | 0.218 | 0.039 | 0.179 | 0.040 | 0.140 | 0.349 | 0.082 | 0.270 | 0.267 | BDL |
| HN-69 | 8.03 | 117 | 1.51 | 9.26 | 77.2 | 0.400 | 0.003 | 0.397 | 0.142 | 0.255 | 0.129 | 0.048 | 0.080 | 0.081 | BDL |
| HN-70 | 7.91 | 118 | 0.803 | 12.2 | 101 | 0.345 | 0.189 | 0.156 | 0.140 | 0.016 | 0.126 | 0.042 | 0.080 | 0.084 | BDL |
| HN-71 | 8.10 | 180 | 0.009 | 18.5 | 154 | 0.062 | 0.043 | 0.019 | 0.019 | BDL | 0.472 | 0.119 | 0.350 | 0.353 | BDL |
| A-73 | 8.24 | 348 | 2.65 | 13.9 | 116 | 0.081 | 0.003 | 0.078 | 0.008 | 0.070 | 0.321 | 0.128 | 0.190 | 0.194 | BDL |
| A-77 | 7.81 | 290 | 1.41 | 7.55 | 62.9 | 0.040 | NA | 0.040 | 0.019 | 0.021 | 0.206 | 0.063 | 0.140 | 0.143 | BDL |
| A-78 | 7.99 | 143 | 0.733 | 6.96 | 58.0 | 0.167 | 0.051 | 0.115 | 0.031 | 0.084 | 0.190 | 0.054 | 0.140 | 0.136 | BDL |
| A-79 | 7.93 | 147 | 0.665 | 8.28 | 69.0 | 0.146 | 0.065 | 0.080 | 0.036 | 0.045 | 0.212 | 0.060 | 0.150 | 0.152 | BDL |
| A-81 | 7.81 | 95.0 | 0.806 | 5.83 | 48.6 | 0.196 | 0.111 | 0.085 | 0.039 | 0.046 | 0.246 | 0.055 | 0.190 | 0.192 | BDL |
| A-83 | 7.05 | 104 | 1.59 | 7.69 | 64.1 | 0.034 | 0.001 | 0.033 | 0.015 | 0.019 | 0.133 | 0.051 | 0.080 | 0.082 | BDL |
| A-84 | 7.14 | 73.0 | 0.506 | 5.51 | 45.9 | 0.144 | NA | 0.144 | 0.019 | 0.125 | 0.093 | 0.018 | 0.080 | 0.075 | BDL |
| A-85 | 7.41 | 67.0 | 1.010 | 5.76 | 48.0 | 0.148 | 0.098 | 0.050 | 0.014 | 0.036 | 0.091 | 0.030 | 0.060 | 0.060 | BDL |
| A-86 | 7.35 | 89.0 | 0.615 | 6.47 | 53.9 | 0.046 | 0.001 | 0.045 | 0.012 | 0.034 | 0.119 | 0.029 | 0.090 | 0.090 | BDL |
| A-88 | 8.00 | 32.0 | 1.03 | 2.53 | 21.1 | 0.262 | 0.186 | 0.075 | 0.018 | 0.058 | 0.047 | 0.028 | 0.020 | 0.019 | BDL |
| A-90 | 7.61 | 55.0 | 0.646 | 4.43 | 36.9 | 0.080 | NA | 0.079 | 0.018 | 0.061 | 0.068 | 0.029 | 0.040 | 0.039 | BDL |
| A-91 | 7.11 | 435 | 1.31 | 4.32 | 36.0 | 0.103 | NA | 0.103 | 0.026 | 0.077 | 0.398 | 0.104 | 0.290 | 0.295 | BDL |
| S-92 | 7.58 | 106 | 1.69 | 6.40 | 53.4 | 0.523 | 0.172 | 0.351 | 0.131 | 0.220 | 0.152 | 0.052 | 0.100 | 0.099 | BDL |
| S-93 | 7.39 | 58.0 | 0.376 | 2.72 | 22.7 | 0.161 | 0.004 | 0.157 | 0.019 | 0.137 | 0.103 | 0.029 | 0.070 | 0.074 | BDL |
| S-95 | 7.31 | 585 | 3.55 | 39.3 | 328 | 0.978 | NA | 0.978 | 0.933 | 0.045 | 0.908 | 0.239 | 0.670 | 0.669 | BDL |
| S-96 | 8.20 | 147 | 2.22 | 14.3 | 119 | 0.362 | 0.158 | 0.203 | 0.203 | BDL | 0.236 | 0.085 | 0.150 | 0.151 | BDL |
| S-99 | 8.11 | 193 | 1.08 | 13.6 | 114 | 0.059 | NA | 0.059 | 0.032 | 0.027 | 0.567 | 0.145 | 0.420 | 0.421 | BDL |
| S-100 | 7.54 | 314 | 1.93 | 10.0 | 83.3 | 0.231 | 0.154 | 0.077 | 0.035 | 0.043 | 0.384 | 0.106 | 0.280 | 0.278 | BDL |
| S-102 | 7.62 | 73.0 | 0.516 | 5.20 | 43.3 | 0.108 | NA | 0.108 | 0.058 | 0.050 | 0.107 | 0.029 | 0.080 | 0.078 | BDL |
| S-104 | 8.34 | 238 | 2.93 | 21.6 | 180 | 1.19 | 0.066 | 1.13 | 1.08 | 0.051 | 0.316 | 0.070 | 0.250 | 0.246 | BDL |
| S-107 | 7.91 | 237 | 3.52 | 21.9 | 182 | 0.741 | 0.108 | 0.633 | 0.615 | 0.018 | 0.283 | 0.083 | 0.200 | 0.200 | BDL |
| S-109 | 7.95 | 128 | 2.19 | 10.5 | 87.4 | 0.074 | 0.045 | 0.029 | 0.022 | 0.006 | 0.137 | 0.055 | 0.080 | 0.082 | BDL |
| S-111 | 8.60 | 113 | 0.922 | 9.88 | 82.3 | 0.134 | NA | 0.134 | 0.037 | 0.097 | 0.137 | 0.042 | 0.100 | 0.095 | BDL |

BDL: Below detection limit, NA: Data not available

Table B2. Concentration (µg kg^-1^) of iodine species (Org I_sol_ and Inorg I_sol_) in I_sol_; the proportion of organic iodine (%Org I_sol_) and inorganic iodine (%Inorg I_sol_) as a percentage of Total I_sol_ and the proportions of iodide (%I^-^_sol_) and iodate (%IO_3_^-^_sol_) in Inorg I_sol_

| **District** | **Sample code** | **Org I_sol_** | **Inorg I_sol_** |  | **%Org I_sol_** | **%Inorg I_sol_** | **%I^-^_sol_** | **%IO_3_^-^_sol_** |  |
| --- | --- | --- | --- | --- | --- | --- | --- | --- | --- |
|  |  | **(µg kg^-1^)** | |  | **(%)** | | | | |
| Gilgit | G01 | 16.4 | 1.18 |  | 93.3 | 6.73 | 97.6 | 2.42 |  |
|  | G02 | 14.2 | 0.134 |  | 99.1 | 0.94 | 89.4 | 10.6 |  |
|  | G03 | 9.82 | 0.107 |  | 98.9 | 1.08 | 57.3 | 42.7 |  |
|  | G04 | 9.27 | 0.166 |  | 98.2 | 1.76 | 60.7 | 39.3 |  |
|  | G05 | 14.3 | 0.046 |  | 99.7 | 0.32 | 54.9 | 45.1 |  |
| Diamer | D06 | 21.5 | 0.440 |  | 98.0 | 2.01 | 100 | 0.00 |  |
|  | D07 | 20.9 | 2.15 |  | 90.7 | 9.32 | 92.9 | 7.07 |  |
|  | D08 | 22.0 | 2.66 |  | 89.2 | 10.8 | 86.3 | 13.7 |  |
|  | D09 | 14.9 | 0.450 |  | 97.1 | 2.92 | 17.5 | 82.5 |  |
|  | D10 | 10.9 | 0.341 |  | 97.0 | 3.03 | 38.3 | 61.7 |  |
| Hunza-N | N11 | 13.7 | 0.155 |  | 98.9 | 1.12 | 82.9 | 17.1 |  |
|  | H12 | 15.0 | 0.122 |  | 99.2 | 0.81 | 47.0 | 53.0 |  |
|  | N13 | 12.3 | 0.072 |  | 99.4 | 0.58 | 33.6 | 66.4 |  |
|  | H14 | 8.50 | 0.129 |  | 98.5 | 1.49 | 46.3 | 53.7 |  |
|  | H15 | 8.04 | 0.113 |  | 98.6 | 1.38 | 22.1 | 77.9 |  |
|  | N16 | 8.77 | 0.123 |  | 98.6 | 1.39 | 55.7 | 44.3 |  |
| Astor | A17 | 7.27 | 0.046 |  | 99.4 | 0.63 | 51.9 | 48.1 |  |
|  | A18 | 8.30 | 0.076 |  | 99.1 | 0.90 | 65.4 | 34.6 |  |
|  | A19 | 7.22 | 0.174 |  | 97.6 | 2.35 | 95.3 | 4.66 |  |
|  | A20 | 9.93 | 0.155 |  | 98.5 | 1.54 | 37.5 | 62.5 |  |
|  | A21 | 6.58 | 0.072 |  | 98.9 | 1.08 | 41.9 | 58.1 |  |
| Skardu | S22 | 15.7 | 0.142 |  | 99.1 | 0.89 | 100 | 0.00 |  |
|  | S23 | 9.29 | 0.193 |  | 98.0 | 2.04 | 2.92 | 97.1 |  |
|  | S24 | 14.4 | 0.162 |  | 98.9 | 1.11 | 80.1 | 19.9 |  |
|  | S25 | 8.19 | 0.109 |  | 98.7 | 1.32 | 92.6 | 7.35 |  |
|  | S26 | 8.24 | 0.142 |  | 98.3 | 1.69 | 89.5 | 10.5 |  |

Table B3. Concentration (µg kg^-1^) of iodine species (Org I_ads_ and Inorg I_ads_) in I_ads_; the proportion of organic iodine (%Org I_ads_) and inorganic iodine (%Inorg I_ads_) as a percentage of Total I_ads_ and the proportions of iodide (%I^-^_ads_) and iodate (%IO_3_^-^_ads_) in Inorg I_ads_

| **District** | **Sample code** | **Org I_ads_** | **Inorg I_ads_** |  | **%Org I_ads_** | **%Inorg I_ads_** | **%I^-^_ads_** | **%IO_3_^-^_ads_** |
| --- | --- | --- | --- | --- | --- | --- | --- | --- |
|  |  | **(µg kg^-1^)** | |  | **(%)** | | | |
| Gilgit | G01 | 17.5 | 2.74 |  | 86.5 | 13.5 | 96.0 | 3.98 |
|  | G02 | 17.3 | 2.17 |  | 88.9 | 11.1 | 87.7 | 12.3 |
|  | G03 | 7.70 | 1.17 |  | 86.8 | 13.2 | 83.8 | 16.2 |
|  | G04 | 12.1 | 0.51 |  | 96.0 | 4.01 | 82.7 | 17.3 |
|  | G05 | 17.8 | 1.07 |  | 94.3 | 5.70 | 95.6 | 4.45 |
| Diamer | D06 | 16.6 | 2.84 |  | 85.4 | 14.6 | 74.0 | 26.0 |
|  | D07 | 22.4 | 6.20 |  | 78.3 | 21.7 | 97.7 | 2.29 |
|  | D08 | 22.4 | 6.65 |  | 77.1 | 22.9 | 86.7 | 13.3 |
|  | D09 | 17.1 | 1.64 |  | 91.3 | 8.73 | 51.8 | 48.2 |
|  | D10 | 9.24 | 0.92 |  | 91.0 | 9.03 | 80.2 | 19.8 |
| Hunza-N | N11 | 17.1 | 0.77 |  | 95.7 | 4.33 | 88.3 | 11.7 |
|  | H12 | 14.5 | 0.96 |  | 93.8 | 6.22 | 75.1 | 24.9 |
|  | N13 | 16.2 | 0.67 |  | 96.0 | 3.99 | 96.3 | 3.66 |
|  | H14 | 8.99 | 0.63 |  | 93.4 | 6.58 | 91.2 | 8.79 |
|  | H15 | 9.93 | 0.23 |  | 97.7 | 2.31 | 40.0 | 60.0 |
|  | N16 | 10.2 | 0.45 |  | 95.8 | 4.22 | 79.2 | 20.8 |
| Astor | A17 | 9.97 | 0.34 |  | 96.7 | 3.34 | 70.8 | 29.2 |
|  | A18 | 10.4 | 1.77 |  | 85.5 | 14.5 | 97.9 | 2.10 |
|  | A19 | 8.44 | 2.02 |  | 80.7 | 19.3 | 97.3 | 2.71 |
|  | A20 | 13.1 | 1.52 |  | 89.6 | 10.4 | 95.6 | 4.44 |
|  | A21 | 10.5 | 1.27 |  | 89.2 | 10.8 | 94.3 | 5.73 |
| Skardu | S22 | 17.6 | 0.36 |  | 98.0 | 2.01 | 88.1 | 11.9 |
|  | S23 | 15.3 | 0.61 |  | 96.2 | 3.82 | 95.4 | 4.65 |
|  | S24 | 17.6 | 2.34 |  | 88.3 | 11.7 | 98.3 | 1.67 |
|  | S25 | 10.1 | 0.38 |  | 96.3 | 3.66 | 72.6 | 27.4 |
|  | S26 | 11.1 | 0.75 |  | 93.7 | 6.28 | 71.3 | 28.7 |

Table B4. Concentration (µg kg^-1^) of Se species (Org Se_sol_ and Inorg Se_sol_) in Se_sol_; the proportion of organic Se (%Org Se_sol_) and inorganic Se (%Inorg Se_sol_) as a percentage of Total Se_sol_ and the proportions of Selenite (%Se_sol_^IV^) and selenate (%Se_sol_^VI^) in Inorg Se_sol_

| **District** | **Sample code** | **Org Se_sol_** | **Inorg Se_sol_** |  | **%Org Se_sol_** | **%Inorg Se_sol_** | **%Se_sol_^IV^** | **%Se_sol_^VI^** |
| --- | --- | --- | --- | --- | --- | --- | --- | --- |
|  |  | **(µg kg^-1^)** | |  | **(%)** | | | |
| Gilgit | G01 | 1.07 | 1.05 |  | 50.5 | 49.5 | 95.8 | 4.17 |
|  | G02 | 1.22 | 0.773 |  | 61.3 | 38.7 | 97.4 | 2.55 |
|  | G03 | 3.49 | 0.953 |  | 78.6 | 21.4 | 96.2 | 3.85 |
|  | G04 | 0.951 | 0.699 |  | 57.6 | 42.4 | 100 | 0.00 |
|  | G05 | 1.45 | 1.32 |  | 52.3 | 47.7 | 100 | 0.00 |
| Diamer | D06 | 1.18 | 0.923 |  | 56.2 | 43.8 | 95.7 | 4.29 |
|  | D07 | 1.58 | 1.84 |  | 46.2 | 53.8 | 100 | 0.00 |
|  | D08 | 1.03 | 0.757 |  | 57.6 | 42.4 | 97.0 | 3.00 |
|  | D09 | 1.13 | 0.201 |  | 84.9 | 15.1 | 96.8 | 3.15 |
|  | D10 | 0.895 | 0.171 |  | 83.9 | 16.1 | 100 | 0.00 |
| Hunza-N | N11 | 2.40 | 2.71 |  | 47.0 | 53.0 | 97.0 | 2.96 |
|  | H12 | 1.77 | 0.308 |  | 85.2 | 14.8 | 95.1 | 4.86 |
|  | N13 | 4.00 | 2.52 |  | 61.4 | 38.6 | 98.8 | 1.23 |
|  | H14 | 2.81 | 1.84 |  | 60.4 | 39.6 | 100 | 0.00 |
|  | H15 | 1.71 | 0.491 |  | 77.7 | 22.3 | 97.6 | 2.44 |
|  | N16 | 2.07 | 0.444 |  | 82.4 | 17.6 | 97.2 | 2.80 |
| Astor | A17 | 1.39 | 0.183 |  | 88.3 | 11.7 | 97.7 | 2.27 |
|  | A18 | 1.14 | 0.499 |  | 69.5 | 30.5 | 98.7 | 1.26 |
|  | A19 | 0.618 | 0.801 |  | 43.6 | 56.4 | 98.2 | 1.83 |
|  | A20 | 1.10 | 0.123 |  | 89.9 | 10.1 | 83.7 | 16.3 |
|  | A21 | 0.816 | 0.135 |  | 85.8 | 14.2 | 87.5 | 12.5 |
| Skardu | S22 | 2.21 | 1.46 |  | 60.3 | 39.7 | 100 | 0.00 |
|  | S23 | 1.18 | 0.682 |  | 63.5 | 36.5 | 96.9 | 3.12 |
|  | S24 | 1.98 | 1.84 |  | 51.8 | 48.2 | 99.7 | 0.31 |
|  | S25 | 1.85 | 0.971 |  | 65.5 | 34.5 | 98.1 | 1.94 |
|  | S26 | 1.10 | 0.293 |  | 79.0 | 21.0 | 99.1 | 0.92 |

Table B5. Concentration (µg kg^-1^) of Se species (Org Se_ads_ and Inorg Se_ads_) in Se_ads_; the proportion of organic Se (%Org Se_ads_) and inorganic Se (%Inorg Se_ads_) as a percentage of Total Se_ads_ and the proportions of Selenite (%Se_ads_^IV^) and selenate (%Se_ads_^VI^) in Inorg Se_ads_

| **District** | **Sample code** | **Org Se_ads_** | **Inorg Se_ads_** |  | **%Org Se_ads_** | **%Inorg Se_ads_** | **%Se_ads_^IV^** | **%Se_ads_^VI^** |
| --- | --- | --- | --- | --- | --- | --- | --- | --- |
|  |  | **(µg kg^-1^)** | |  | **(%)** | | | |
| Gilgit | G01 | 0.506 | 2.58 |  | 16.4 | 83.6 | 99.1 | 0.887 |
|  | G02 | 0.813 | 2.47 |  | 24.8 | 75.2 | 99.0 | 0.988 |
|  | G03 | 1.85 | 2.41 |  | 43.4 | 56.6 | 99.7 | 0.288 |
|  | G04 | 1.07 | 1.18 |  | 47.4 | 52.6 | 99.3 | 0.690 |
|  | G05 | 1.29 | 2.80 |  | 31.5 | 68.5 | 100 | 0.268 |
| Diamer | D06 | 1.23 | 1.01 |  | 55.0 | 45.0 | 99.1 | 0.867 |
|  | D07 | 1.25 | 3.36 |  | 27.1 | 72.9 | 100 | 0.438 |
|  | D08 | 0.479 | 1.44 |  | 25.0 | 75.0 | 99.0 | 1.001 |
|  | D09 | 0.898 | 0.358 |  | 71.5 | 28.5 | 99.2 | 0.843 |
|  | D10 | 0.685 | 0.311 |  | 68.8 | 31.2 | 100 | 0.095 |
| Hunza-N | N11 | 0.00 | 7.56 |  | 0.00 | 100 | 99.9 | 0.107 |
|  | H12 | 1.27 | 0.72 |  | 64.1 | 35.9 | 98.4 | 1.55 |
|  | N13 | 0.127 | 7.70 |  | 1.63 | 98.4 | 99.8 | 0.226 |
|  | H14 | 0.673 | 4.10 |  | 14.1 | 85.9 | 99.4 | 0.591 |
|  | H15 | 0.748 | 0.875 |  | 46.1 | 53.9 | 99.4 | 0.648 |
|  | N16 | 1.14 | 0.838 |  | 57.7 | 42.3 | 98.8 | 1.15 |
| Astor | A17 | 0.876 | 0.255 |  | 77.4 | 22.6 | 97.6 | 2.39 |
|  | A18 | 0.877 | 0.595 |  | 59.6 | 40.4 | 97.2 | 2.77 |
|  | A19 | 0.593 | 1.11 |  | 34.9 | 65.1 | 99.1 | 0.87 |
|  | A20 | 1.105 | 0.151 |  | 87.9 | 12.1 | 94.4 | 5.60 |
|  | A21 | 0.813 | 0.175 |  | 82.3 | 17.7 | 94.1 | 5.88 |
| Skardu | S22 | 0.604 | 1.61 |  | 27.3 | 72.7 | 99.0 | 1.04 |
|  | S23 | 0.527 | 1.49 |  | 26.2 | 73.8 | 99.1 | 0.90 |
|  | S24 | 0.00 | 5.21 |  | 0.00 | 100 | 99.8 | 0.245 |
|  | S25 | 0.122 | 2.39 |  | 4.86 | 95.1 | 99.4 | 0.617 |
|  | S26 | 0.355 | 0.589 |  | 37.6 | 62.4 | 97.2 | 2.81 |
